# Determination of End-Group Functionality of Propylene Oxide-Based Polyether Polyols Recovered from Polyurethane Foams by Chemical Recycling

**DOI:** 10.1021/acs.macromol.3c00087

**Published:** 2023-04-20

**Authors:** Blaž Zdovc, Maja Grdadolnik, David Pahovnik, Ema Žagar

**Affiliations:** Department of Polymer Chemistry and Technology, National Institute of Chemistry, Hajdrihova 19, Ljubljana SI-1000, Slovenia

## Abstract

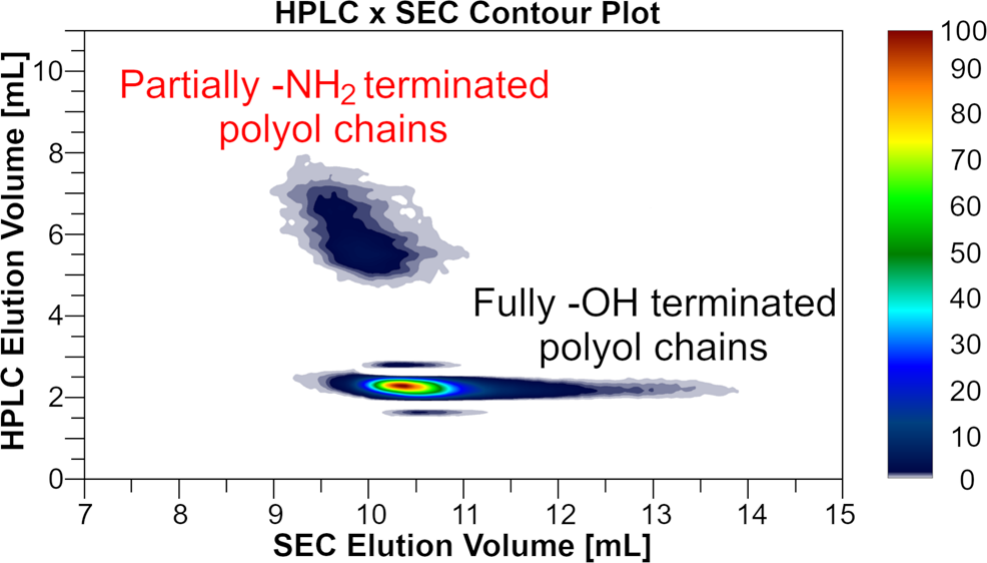

Chemical recycling of polyurethane foams (PUFs) leads to partially aromatic, amino-functionalized polyol chains when the urethane groups in the PUF structure are incompletely degraded. Since the reactivity of amino and hydroxyl groups with isocyanate groups is significantly different, information on the type of the end-group functionality of recycled polyols is important to adjust the catalyst system accordingly to produce PUFs from recycled polyols of suitable quality. Therefore, we present here a liquid adsorption chromatography (LAC) method using a SHARC 1 column that separates polyol chains according to their end-group functionality based on their ability to form hydrogen bonds with the stationary phase. To correlate end-group functionality of recycled polyol with chain size, LAC was coupled with size-exclusion chromatography (SEC) to form a two-dimensional liquid chromatography system. For accurate identification of peaks in LAC chromatograms, the results were correlated with those obtained by characterization of recycled polyols using nuclear magnetic resonance, matrix-assisted laser desorption/ionization time-of-flight mass spectrometry, and SEC coupled with a multi-detection system. The developed method allows the quantification of fully hydroxyl-functionalized chains in recycled polyols using an evaporative light scattering detector and appropriate calibration curve.

## 1 Introduction

Polyurethane foams (PUFs) account for more than 55% of total polyurethane production.^1,2^ Flexible PUFs are used in the automotive industry, carpets, cushions, beds, and furniture, while rigid PUFs are commonly used in building and appliance insulation and packaging.^3−6^ As the global market for PUF production is expected to increase to 12.74 million tons by 2024, PUF waste is also expected to increase.^1,7,8^ For this reason, recycling of PUF waste is important from environmental and economic perspectives.

Because PUFs are thermoset polymers with a cross-linked structure and good thermal stability, mechanical recycling is not a suitable method for PUF waste management.^3,9^ Therefore, chemical recycling has become an important research field in recent years. Chemical recycling is based on the cleavage of urethane groups in the PUF structure to generate the recycled polyol (RP) and residual hard segments.^6,10^ Chemical recycling of PUFs by hydrolysis,^11,12^ glycolysis,^6,10,13−20^ or aminolysis^9,21−23^ results in partially aromatic amino-functionalized polyol chains in the case of incomplete degradation of urethane groups in the PUF structure. It has been shown that the type of end-group functionality^24^ and the presence of the low molar mass, amino-functionalized side products in RPs together with other impurities soluble in RPs^10,18,25^ prevent the synthesis of new PUFs exclusively from RPs. Therefore, only part of the virgin polyol (VP) can be replaced by RP in the PUF formulation to obtain PUFs with suitable mechanical properties.^10,18,24^ Since aromatic amino groups react significantly faster with diisocyanates than hydroxyl groups^3,26^ knowledge of the end-group functionality of RPs is important to modify the catalytic system and appropriately adjust the relative kinetics of foaming and cross-linking reactions with the aim of producing high-performance PUFs from RPs.

Characterization methods to evaluate the quality of RPs include conventional determination of hydroxyl number, acid value, and water content.^10,15,16,19,24,27^ Size-exclusion chromatography (SEC)^9,10,13,16,24,27^ or viscosity^9,14,19^ is used to determine the RP chain length, while structure and purity of RPs are evaluated by nuclear magnetic resonance (NMR)^10,17,20,24,27^ and Fourier transform infrared spectroscopy (FTIR).^15−17,20^ The presence of low molar mass, amino-functionalized side products, aromatic diamines, and other impurities such as residual catalyst and reagent soluble in RPs can be assessed by NMR,^10,17,20,24,27^ SEC,^9,10,13,16,24^ gas chromatography,^10,12,14,28^ and LC on a reversed^13,22^ or mixed-mode^29^ stationary phase. Despite the large number of characterization techniques used to evaluate the quality of RPs, methods to determine end-group functionality of RPs are lacking.

The polyols used for PUF synthesis are star-shaped, hydroxyl-functionalized propylene oxide (PO)-based polyether polyols. Although the literature on the characterization of ethylene oxide (EO)-based polyols is extensive, there are only few methods for characterizing hydrophobic PO-based polyether polyols, i.e., reversed-phase or normal-phase LC and SEC, which provide information on the size of the polyol chains.^30−33^ When PO-based polyols are end-functionalized with amino groups, size-separation by LC techniques is even more challenging because basic amino groups show strong interaction with conventional column matrices.^34^ Therefore, either the use of additives in the mobile phase such as trifluoroacetic acid (TFA) or the derivatization of the amino groups to amides is necessary.^34^ However, even in these cases, the use of a mass-selective detector for the unambiguous identification of individual peaks in the chromatograms is inevitable since the retention of individual polyol species in the column depends not only on the type of end groups but also on the molar mass and chemical composition of the copolyether polymers. Very recently, polypropylene oxides (PPOs) were simultaneously separated according to the number of hydroxyl end-groups, which depends on the initiator used for PPO synthesis, and the molar mass by 2D-LC.^35^

Here, we present a detailed characterization of RPs chemically recovered from flexible PUFs, which were synthesized from the homo- or copolymeric polyether polyol, the isomeric mixture of toluene diisocianate (TDI) and water as a latent-foaming agent. To this end, we have developed an isocratic LAC method that separates the components of RPs according to end-group functionality and allows quantification of fully hydroxyl-functionalized polyol chains in RPs. To accurately identify peaks in LAC chromatograms and correlate the end-group functionality of eluted RP species with their hydrodynamic volume, LAC was coupled on-line with SEC to form a LAC × SEC 2D-LC system (Figure S1). The results of LAC and LAC × SEC 2D-LC are supported by the results of structure and molar mass characterization of RPs by NMR, MALDI-TOF MS, and SEC coupled to a multi-detection system.

## 2 Experimental Section

### 2.1 Materials and Chemicals

The VPs Alcupol F-5611 and Alcupol F-4811 (labeled as VP4811 and VP5611, respectively) and the PUFs prepared from these polyols were supplied by Repsol S.A. VP5611 is a trifunctional homopolyether polyol consisting of PO repeating units attached to a glycerol core. It has a hydroxyl number of 56 mg KOH g^–1^ and an average molar mass of 3.0 kg mol^–1^. VP4811 is a trifunctional copolyether polyol consisting of PO and 12 mol % EO repeating units attached to a glycerol core. It has a hydroxyl number of 48 mg KOH g^–1^ and an average molar mass of 3.5 kg mol^–1^. Both ALCUPOL polyols contain a mixture of isomers (3,5-di-*trans*-butyl-4-hydroxyphenyl)propionate of C7–9-alkyl as a phenolic antioxidant (CAS 125643-61-0; Additive 1) and benzenamine, *N*-phenyl-, reaction products with 2,4,4-trimethylpentene as an amine antioxidant (CAS 68411-46-1; Additive 2). PUF5611 and PUF4811 were synthesized from VP5611 (66.1 wt % polyol per PUF) and VP4811 (65.9 wt % polyol per PUF), respectively, a mixture of 2,4-TDI and 2,6-TDI with an isomer ratio of 80/20 (TDI8020, TDI index of 107), and water as the foaming agent to chemically generate CO_2_. Kosmos 29 (tin(II) octoate), TEGOAMIN 33, and TEGOAMIN BDE were used as catalysts, and silicone TEGOSTAB BF 2370 was used as a surfactant to control cell size and opening. The chemical degradation procedures for PUFs with detailed reaction conditions to recover RPs with different contents of aromatic amino end-groups are described in Supporting Information. The samples are designated RP5611-XX.X or RP4811-XX.X, where RP stands for recycled polyol and the numbers 5611 and 4811 stand for PPO-based and P(PO-*co*-EO)-based polyols, respectively, while the numbers after the hyphen (XX.X) indicate the content of residual aromatic amino end-groups in RP as determined by ^1^H NMR according to eq S1.

Tris(2-aminoethyl)amine (TREN; Sigma-Aldrich, Germany) and hyperbranched polyethylenimine with a number-average molar mass of 600 g mol^–1^ (PEI-600; Sigma-Aldrich, Germany) were used as degradation reagents. Acetonitrile (ACN; ≥99.9%; Riedel de Haën, Germany), methanol (MeOH; ≥99.9%; Merck, Germany), formic acid (FA; ≥98%; Fluka, Germany), ammonium formate (AmFm; ≥97%; Fluka, Germany), and MQ-water (MQ) with 18.2 MΩ resistivity were used for HPLC experiments, while MeOH and polyethylene glycol (PEG) with a weight-average molar mass of 4.0 kg mol^–1^ were used in SEC experiments. Deuterated dimethyl sulfoxide (DMSO-*d*_6_; Euriso Top, Germany) and TFA (99%; Aldrich, Germany) were used for ^1^H NMR experiments. Hydrochloric acid (HCl; Euriso Top, Germany), ethyl acetate (EtOAc; Honeywell, USA), and MQ-water (MQ) were used for purification of RPs. Imidazole (Sigma-Aldrich, Germany), pyridine (Sigma-Aldrich, Germany), phenolphthalein (Merck, Germany), phthalic anhydride (Merck, Germany), potassium hydrogen phthalate (KHP; Acros Organics, USA), sodium hydroxide (NaOH; Honeywell, USA), and ethanol (EtOH; Carlo Erba, Italy) were used to determine the hydroxyl number and acid value of RPs. Aquastar water standard 0.01% (Supelco, Germany), Aquastar CombiCoulomat reagent (Supelco, Germany), and chloroform (CHCl_3_; Honeywell, Fluka) were used to determine water content in RPs. Tetrahydrofuran (THF, p.a.; Merck, Germany), 2,5-dihydroxybenzoic acid (≥99.0%; Aldrich, Germany), sodium trifluoroacetate (≥98.0%; Aldrich, Germany), and poly(methyl methacrylate) standards (PMMA; MALDI validation set, Fluka Analytical) were used to perform MALDI-TOF mass spectrometry experiments. All chemicals were used as received.

### 2.2 LAC

Separation of polyols according to end-group functionality was performed with isocratic LAC on a SiELC SHARC 1 column (4.6 mm × 150 mm, 100 Å, 5 μm; SiELC Techonologies, USA) at a temperature of 25 °C maintained with a thermostatted oven. An HPLC pump (Agilent 1260, Agilent Technologies, USA) delivered the mobile phase at a nominal flow rate of 1 mL/min. The homopolymeric polyols were separated according to end-group functionality using a mobile phase composed of 75% ACN with 3.00 vol % FA and 0.048 vol % MQ and 25% MeOH with 0.1 mg mL^–1^ AmFm, while the copolymeric polyols were separated using a mobile phase composed of 75% ACN with 6.00 vol % FA and 0.048 vol % MQ and 25% MeOH with 0.1 mg mL^–1^ AmFm. An ultraviolet (UV) detector operating at a wavelength of 283 nm and an ELS detector 1260 Infinity (both Agilent Technologies, USA) connected in series were used to detect the eluted species.

### 2.3 LAC × SEC 2D-LC

In the first dimension (LAC), the experiments were performed on the same SiELC SHARC 1 column as in LAC. In the second dimension (SEC), a GRAM high-speed column (20 mm × 50 mm, 100 Å, 10 μm, Polymer Standards Service, PSS GmbH, Germany) was used, which has a broad pore size distribution and covers the molar mass range from 3 × 10^2^ to 6 × 10^4^ g mol^–1^. For the homo- and copolymeric polyols, the composition of the mobile phase in both LC dimensions of the 2D-LC was the same as in the LAC experiments. The flow rates in the first (LAC) and second (SEC) LC dimensions were set at 0.025 and 3 mL/min, respectively. The 2D-LC experiments were performed at 25 °C. The injected mass of the samples in the first dimension was typically 20 μg. The same detectors were used for detection under the same conditions as in the LAC experiments.

### 2.4 Quantification of Fully Hydroxyl-Functionalized Polyol Chains in the RPs

Quantification of fully hydroxyl-functionalized polyol chains in the RPs was performed using a calibration curve representing the ELS detector response as a function of the VP concentration. For construction of the calibration curve, eight solutions of VP of different concentrations (from 1.00 to 0.44 mg mL^–1^) were prepared. Each standard solution was injected onto the column in triplicate to obtain the average surface area under the VP peak. The RP solutions were prepared by dissolving the samples in the mobile phase at a concentration of 1 mg mL^–1^ and stirring overnight. The injection volume of RP was the same as in the calibration experiments. From the area under the peak corresponding to a fully hydroxyl-functionalized polyol, its weight percentage (wt %) in RP was determined from the corresponding calibration curve.

### 2.5 SEC Coupled to a Multi-Detection System Consisting of UV, Multi-Angle Light Scattering, and Differential Refractive Index Detectors (SEC/UV-MALS-RI)

Molar mass characteristics (molar mass averages and dispersity) of the polyols were determined using SEC coupled to a multi-detection system consisting of a UV (Agilent 1260, Agilent Technologies, USA), a multi-angle light scattering (MALS) photometer with 18 angles (Dawn Neon, Wyatt Technologies, USA), and a refractive index (RI) detector (Optilab, Wyatt Techonologies, USA). Calibration of the 90° light scattering (LS) detector was performed with toluene, while normalization of the other LS detectors was performed with a PEO standard of a weight-average molar mass of 4 kg mol^–1^ and a dispersity of 1.02. Separations were performed on a chromatography system (Agilent 1260, Agilent Technologies, USA) at room temperature using a TSKgel Alpha-2500 SEC column with a precolumn (7.8 mm ID × 30.0 cm L, particle size 7 μm, and exclusion limit 10 kDa; Tosoh Bioscience GmbH, Germany) and MeOH as the mobile phase at a constant flow rate of 0.7 mL min^–1^. The mass of samples injected onto the column was typically 1 × 10^–3^ g, and their concentration in MeOH was typically 1 × 10^–2^ g mL^–1^. The values of the specific refractive index increment (d*n*/d*c*) required to calculate the molar masses of the polyols were determined assuming 100% mass recovery of the samples from the column. The d*n*/d*c* values of VP5611 and VP4811 in MeOH are 0.135 and 0.136 mL g^–1^, respectively, while the d*n*/d*c* of RP depending on the aromatic amino end-group content (d*n*/d*c*)_RP4811-15.9_ is up to 2.9% higher than that of VP4811. Astra 8 software (Wyatt Technologies, USA) was used for data acquisition and analysis.

### 2.6 NMR Spectroscopy

The ^1^H NMR spectra were recorded in DMSO-*d*_6_ with or without the addition of TFA on a Bruker AVANCE NEO 600 MHz instrument (Bruker Corporation, USA). Chemical shifts are given in ppm relative to a DMSO-*d*_6_ residual peak.

### 2.7 MALDI-TOF MS

MALDI-TOF mass spectra of polyols were recorded using a Bruker UltrafleXtreme MALDI-TOF mass spectrometer (Bruker Daltonics, Germany). Polyol samples were dissolved in THF at a concentration of 10 mg mL^–1^. The solutions thus prepared were mixed with a matrix solution of 2,5-dihydroxybenzoic acid in THF (*c* = 30 mg mL^–1^) and a solution of sodium trifluoroacetate in THF (*c* = 10 mg mL^–1^) in a volume ratio of 1:10:3. A 0.4 μL of the mixture was spotted onto a target plate using a dried-droplet method. The mass spectra of the samples were recorded in a reflective positive ion mode. Calibration was performed externally using a mixture of PMMA standards dissolved in THF and covering the measured molecular weight range. The standard mixture was prepared according to the same procedure as for the polyol samples and spotted to the nearest-neighbor positions.

## 3 Results and Discussion

**Figure 1:**
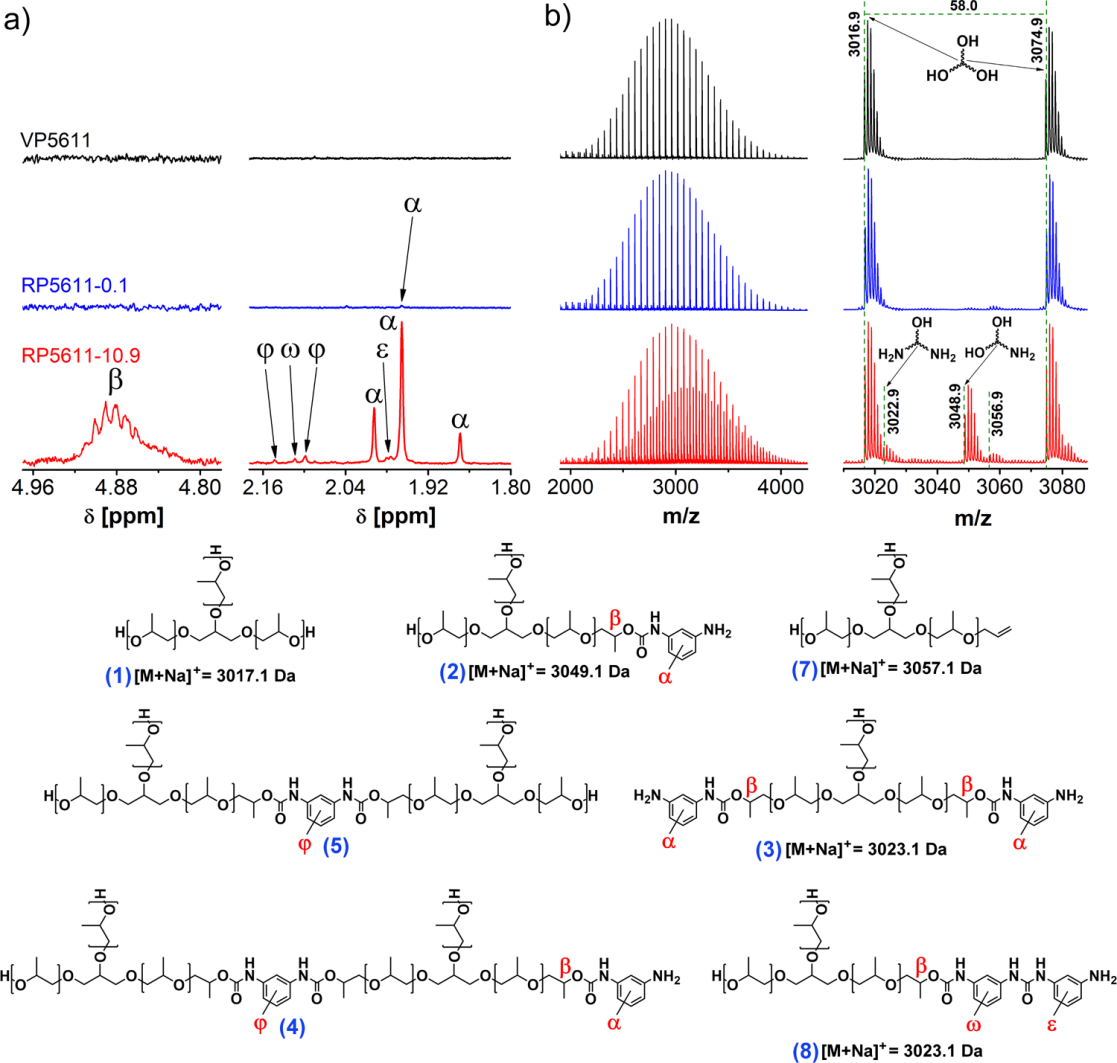
(a) Magnified ^1^H NMR spectra and (b) MALDI-TOF mass spectra of PPO-based VP5611 and corresponding purified RP5611 samples with aromatic amino end-group content of 0.1 and 10.9 mol %. The ^1^H NMR spectra are normalized to the proton signal of the polyol methyl group. Magnified ^1^H NMR spectra in the δ-range 4.78–4.98 ppm were recorded in DMSO-*d*_6_ with added TFA to shift the overlapping amino end-groups toward the lower magnetic field, while spectra in the δ-range 1.80–2.21 ppm were recorded in DMSO-*d*_6_. The measured monoisotopic masses of the different populations, shown in the magnified regions of the mass spectra, agree well with the theoretical monoisotopic masses ionized with the sodium ion [M + Na]^+^ given under the proposed structures.

Most of the existing recycling methods can not completely degrade urethane groups in the PUF structure, which leads to RPs partially functionalized with aromatic amino end-groups and to the possible presence of oligomeric species even after RP purification, as shown by the results of characterization of homo- and copolymeric RPs by MALDI-TOF MS, ^1^H NMR, and SEC/UV-MALS-RI (Figures 1, S2, and S3). By determining the hydroxyl number, we cannot distinguish between hydroxyl and amino end-groups of RPs because phthalic anhydride reagent reacts with both end-group types. The relative content of aromatic amino end-groups and aromatic moieties linking two polyol chains via urethane groups in RPs can be assessed from the ratio of UV to RI detector responses of the polyol peak in SEC/UV-RI chromatograms (Figure S2). While MALDI-TOF MS can determine the exact masses of individual components of homopolymeric PO-based RPs, it cannot distinguish between structural isomers of the same molecular weight (an example is given in Figure 1 for [M + Na]^+^ of 3023.1 Da), nor can individual polyol species be quantified with confidence. ^1^H NMR allows quantification of the residual urethane groups in RPs based on the signal intensity of polyol methine protons (β) near the urethane groups (Figures 1 and S3). The content of aromatic amino end-groups in RPs can be determined from the ^1^H NMR spectra from the signals (α) corresponding to the three isomeric methyl groups of aromatic amino end-groups attached to the polyol chains via the urethane groups and from the aromatic methyl signal (ε) of the urea end-groups (Figures 1 and S3). However, with ^1^H NMR, we cannot distinguish between the mono-, di-, or tri-aromatic amino-functionalized polyol species, so the end-group distribution cannot be assessed. The typical signals of aromatic methyl groups of oligomers present in RPs are denoted with (φ) and correspond to the two aromatic isomeric moieties linking two polyol chains (Figures 1 and S3).

Because RPs are mixtures of different chain lengths with distributed aromatic amino and hydroxyl end-groups in the case of incomplete degradation of urethane groups, our aim was to gain a more detailed insight into the structure of RPs and to determine the content of fully hydroxyl-functionalized polyol chains in RPs. This information can neither be obtained with MALDI-TOF MS, NMR or SEC/UV-MALS-RI nor from the hydroxyl number of RPs. Therefore, we developed an isocratic HPLC method that separates the individual components of RPs according to end-group functionality. The separation was performed by isocratic LAC on a SHARC 1 column without the need to derivatize the polyol amino end-groups. The SHARC 1 column is compatible with ACN and MeOH as weak and strong solvents, respectively, and separates analytes based on the strength of the analyte’s polar interaction with the stationary phase.^36^ Since the polar interaction of amino groups with the stationary phase is stronger than that of the hydroxyl group,^37^ polyol chains are expected to elute from the column according to end-group functionality. The chromatographic behavior of RPs depends not only on the ratio of ACN and MeOH but also on the ratio of AmFm to FA additives in the mobile phase. When the ratio favors AmFm, the separation of the differently functionalized polyol chains in ACN and MeOH (75/25) is unsuccessful (Figure S4). On the other hand, when the ratio favors FA, the amino-functionalized polyol chains are retained in the column longer than fully hydroxyl-functionalized polyol chains (Figure S4). For the separation of the homopolymeric RP5611 according to end-group functionality, a ratio of 3.0 vol % FA in ACN and 0.1 mg mL^–1^ AmFm in MeOH is suitable (Figure S4). However, for the separation of the copolymeric RP4811 (Figure S5), this FA/AmFm ratio is insufficient because the copolymeric polyol also consists of more polar EO repeating units that interact stronger with the column stationary phase (Figure S5). To improve the separation of the copolymeric RP4811 by end-group functionality, a higher amount of FA was required in the mobile phase (Figure S5). The optimal mobile phase composition for the homopolymeric RP on a SHARC 1 column at 25 °C is 75% ACN with 3.0 vol % FA and 0.048 vol % MQ and 25% MeOH with 0.1 mg mL^–1^ AmFm, while for the copolymeric RP, it is necessary to use the FA/AmFm ratio of 6.0 vol % FA in ACN and 0.1 mg mL^–1^ AmFm in MeOH. The optimal mobile phase composition for P(PO-*co*-EO) RP4811 was also tested for the separation of PPO-based polyols (VP5611 and RP5611). In this case, the fully hydroxyl-functionalized polyol chains are well separated from the partially aromatic amino-functionalized polyol chains. However, the resolution between the differently aromatic amino-functionalized polyol chains (peaks 2, 3, 4) is inferior to the optimal composition of the mobile phase for PPO-based polyols.

**Figure 2:**
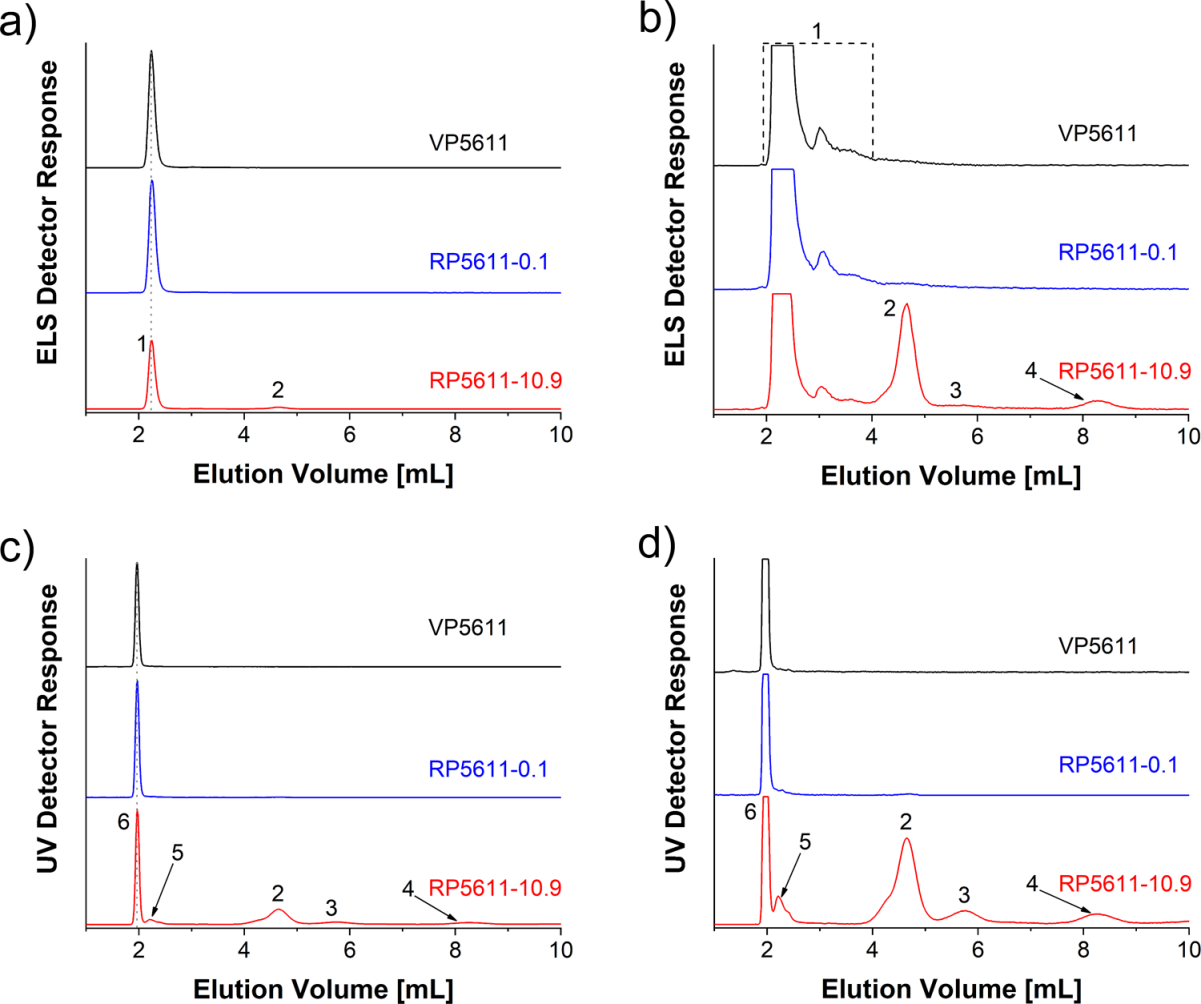
(a) LAC-ELS chromatograms with (b) enlarged peaks and (c) LAC-UV chromatograms with (d) enlarged peaks for PPO-based VP5611 and corresponding purified RP5611 samples containing 0.1 and 10.9 mol % aromatic amino end-groups. Column: SHARC 1, column temperature: 25 °C, mobile phase composition: 75% ACN with 3.0 vol % FA and 0.048 vol % MQ and 25% MeOH with 0.1 mg mL^–1^ AmFm.

Under optimal experimental conditions, the LAC chromatograms recorded with the ELS detector for VP5611 and VP4811 show only one peak (labeled 1) at elution volumes of 2.25 and 2.23 mL, respectively, corresponding to the fully hydroxyl-functionalized polyol chains (Figures 2a,b and S6a, b). The LAC chromatograms of VP5611 and VP4811 recorded with the UV detector at a wavelength of 283 nm show peaks at 1.98 and 1.99 mL, respectively (labeled 6; Figures 2c,d and S6c, d), which correspond to the UV-active phenolic additives present in VPs as antioxidants rather than the VPs, which do not contain any chromophore groups in the structure. This was confirmed by LAC analysis of the pure antioxidant additives, which co-elute with VPs under the same experimental conditions and subsequently by 2D-LC (Figure S7).

The LAC chromatogram of RP5611-10.9 containing 10.9 mol % aromatic amino end-groups, recorded with the ELS detector (Figure 2a,b), shows, in addition to peak 1 for fully hydroxyl-functionalized polyol chains, the additional peaks labeled 2, 3, and 4, all of which are also visible in the UV chromatogram (Figure 2c,d). In addition, the UV chromatogram of RP5611-10.9 shows the low-intensity peak labeled 5 on the right side of peak 6 for antioxidant additives (Figure 2c,d), both of which overlap with the high-intensity peak 1 in the chromatogram recorded with the ELS detector. Similar to RP5611-10.9, the ELS and UV chromatograms of RP4811-15.9 with 15.9 mol % aromatic amino end-groups show several peaks; only the resolution between peaks eluted in the elution volume range of 4.5–9.0 mL is inferior (Figure S6). Since all components of RPs eluted from the HPLC column are detected not only by the universal ELS but also by the UV detector, except the fully hydroxyl-functionalized polyol chains (peak 1), they should contain a chromophore group in the structure.

**Figure 3:**
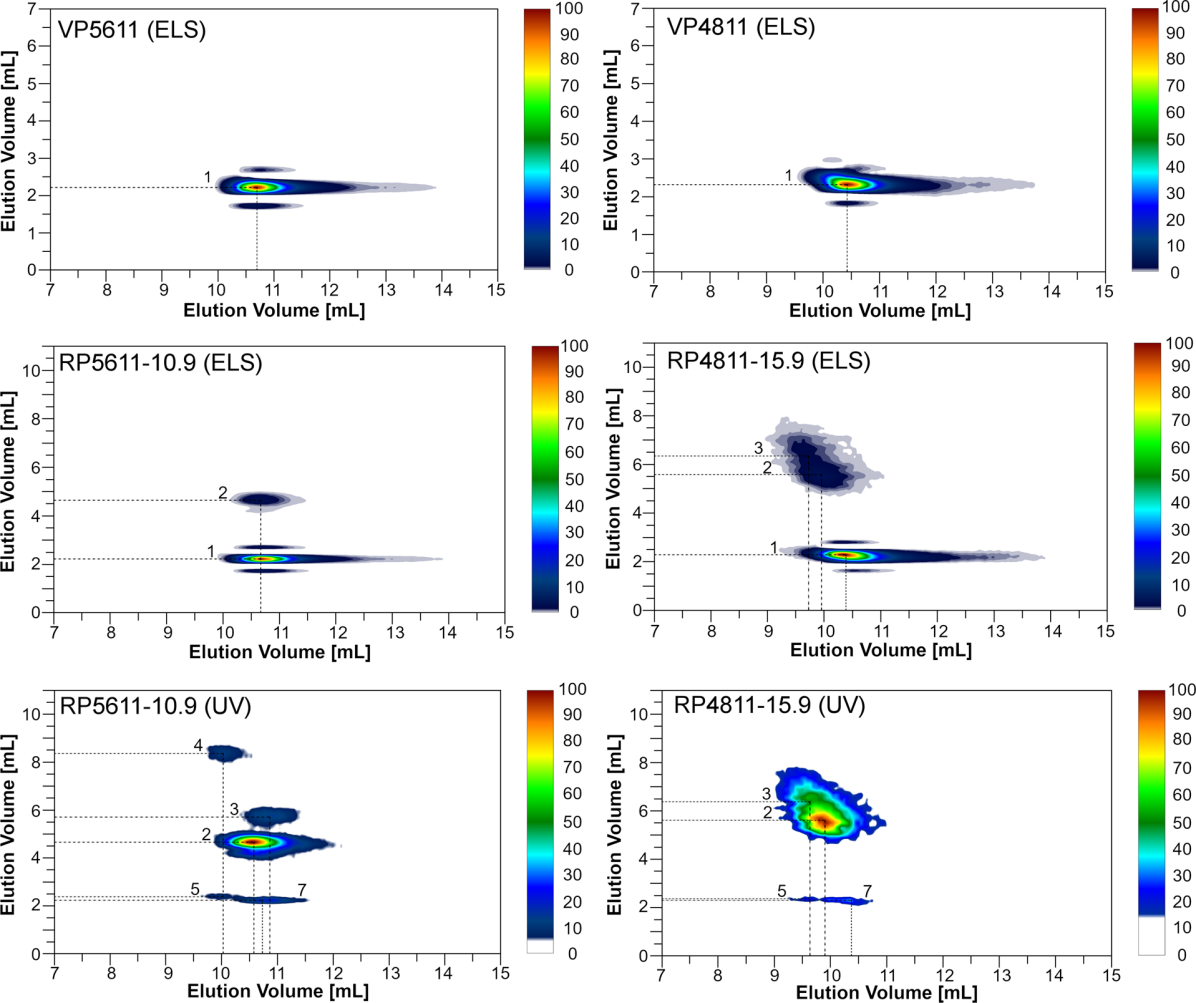
LAC × SEC 2D-LC contour plots of PPO-based VP5611 and purified RP5611-10.9 containing 10.9 mol % aromatic amino end-groups and P(PO-*co*-EO)-based VP4811 and purified RP4811-15.9 containing 15.9 mol % aromatic amino end-groups. 1st-D LAC (*y*-axis): SHARC 1 column; *T* = 25 °C; mobile phase composition for homopolymeric polyols: 75% ACN with 3.0 vol % FA and 0.048 vol % MQ and 25% MeOH with 0.1 mg mL^–1^ AmFm and for copolymeric polyols: 75% ACN with 6.0 vol % FA and 0.048 vol % MQ and 25% MeOH with 0.1 mg mL^–1^ AmFm; flow rate: 0.025 mL min^–1^. 2nd-D SEC (*x*-axis): PSS GRAM high-speed column; *T* = 25 °C; mobile phase the same as in the first dimension; flow rate: 3 mL min^–1^; detector: ELS and UV. The color scale indicates the relative intensity of the ELS or UV detector response.

To accurately identify peaks in LAC chromatograms and correlate the end-group functionality of eluted RP species with their hydrodynamic volume, LAC was coupled on-line with SEC to form a LAC × SEC 2D-LC system (Figure S1). The *y*-axis (1st-D LAC) shows the separation of RP components by end-group functionality, while the *x*-axis (2nd-D SEC) shows the separation of species eluting from the HPLC column by size. The LAC × SEC 2D-LC contour plots recorded by the ELS detector for both VP types show single spots (labeled 1) corresponding to fully hydroxyl-functionalized polyol chains (Figure 3, structure 1 in Figure 1). These spots are not present in the 2D-LC contour plots of VPs recorded with the UV detector, as fully hydroxyl-functionalized polyol chains are not UV-active. Furthermore, the low molar mass antioxidant additives, which almost co-elute with fully hydroxyl-functionalized polyol chains in LAC, completely separate from the polyol in the SEC dimension of 2D-LC and elute near the total permeation limit of the column (elution volume in *x*-axis: 16.6 mL; Figure S7b).

The LAC × SEC 2D-LC (ELS) contour plot of RP5611-10.9 shows a high-intensity spot 1 that elutes in both dimensions in the same elution volume range as spot 1 of VP5611 and is therefore attributed to fully hydroxyl-functionalized polyol chains (Figure 3, structure 1 in Figure 1). The additional spot 2 in the 2D-LC (ELS) is centered in the SEC-dimension (*x*-axis) at a comparable elution volume to spot 1, and therefore, this component should have a comparable molar mass to VP5611. Since spot 2 elutes later from the HPLC column than spot 1 for fully hydroxyl-functionalized polyol chains and is also UV-active (Figure 3), it should represent polyol chains with an aromatic amino end-group, which significantly affects polyol elution behavior in LAC but contributes little to the molar mass of the polyol (only 4.5%). Based on the intensity of spot 2 and the intensity of signals in the MALDI-TOF mass spectrum of RP5611-10.9 (Figure 1b), this component is attributed to the mono-amino-functionalized polyol chains (structures 2 and 8 in Figure 1). Other components of RP5611-10.9 eluted from the HPLC column are not visible in the 2D-LC plot (ELS) due to their small amount and considerable sample dilution during analysis (peaks 3, 4) or overlapping with high-intensity peak 1 (peak 5). However, the components of the RP5611-10.9 that eluted under peaks 2–6 in the HPLC (UV) chromatogram (Figure 2) are all clearly visible in the 2D-LC contour plot recorded with a more sensitive UV detector (Figure 3). Since spot 3 eluted after spot 2 in the LAC dimension, while in the SEC dimension, elution volumes of both spots are comparable to that of VP5611 (Figure 3), and based on the intensity of signals in the MALDI-TOF mass spectrum of RP5611-10.9 (Figure 1b), spot 3 is attributed to the di-amino-functionalized polyol chains (structure 3 in Figure 1). The low-intensity spots 4 and 5 of RP5611-10.9 (Figure 3) elute in the SEC dimension at a comparable elution volume, but in contrast to spots 2 and 3 earlier than fully hydroxyl-functionalized polyol chains in spot 1, indicating the presence of high molar mass components in trace amounts in RP5611-10.9, which should have different end-group functionalities according to their significantly different elution volumes in the LAC dimension. Since spot 5 co-elutes with spot 1 in the LAC dimension, this component cannot be end-functionalized with the amino group, which govern the elution behavior in LAC. For this reason, and based on the results of SEC/UV-MALS-RI (Figure S2a) and ^1^H NMR (Figure 1a), spot 5 most likely represent the fully hydroxyl-functionalized dimer (structure 5 in Figure 1), while spot 4 is assigned to the dimer with at least one amino end-group (structure 4 in Figure 1). The presence of a small amount of dimers in RP5611-10.9 is confirmed in the SEC/UV-MALS-RI chromatograms by the lines representing the molar mass as a function of elution volume, which for RP5611-10.9 deviate slightly from that of VP5611 on the far left side of the polyol peak (6.0–6.25 mL; Figure S2a), which is reflected in its slightly higher molar mass averages (Table 1).

**Table 1:** Weight-Average Molar Mass and Dispersity of VPs and RPs As Determined by SEC/MALS-RI, the Content of Fully Hydroxyl-Functionalized Polyol Chains in RPs As Determined by HPLC and ^1^H NMR, and the Content of Aromatic Amino End-Groups in RPs As Determined by ^1^H NMR

sample	*M*_w_	*Đ*	content of fully hydroxyl-functionalized polyol in RP		–NH_2_ end-group content
	SEC/MALS-RI kg mol^–1^		HPLC wt % per polyol	NMR^b^ mol % per polyol	NMR^a^ mol % per polyol end-groups
VP5611	3.0	1.02	100.0	100.0	/
RP5611-0.1	3.0	1.02	99.9	99.7	0.1
RP5611-10.9	3.2	1.03	68.0	67.3	10.9
RP5611-1.0	3.0	1.02	98.8	97.0	1.0
RP5611-7.5	3.2	1.02	79.4	77.5	7.5
VP4811	3.5	1.02	100.0	100.0	/
RP4811-0.2	3.5	1.02	99.9	99.4	0.2
RP4811-15.9	4.2	1.03	60.8	52.3	15.9
RP4811-6.7	3.7	1.02	82.7	79.9	6.7

a The content of amino end-groups in RP is determined by ^1^H NMR according to eq S1.

b The content of fully hydroxyl-functionalized polyol in RP is determined by ^1^H NMR according to eq S2.

Finally, the 2D-LC (UV) contour plot of RP5611-10.9 also shows the low-intensity spot 7 co-eluting in both dimensions with the UV-inactive, fully hydroxyl-functionalized polyol. MALDI-TOF MS (Figure 1b) and ^1^H NMR results (δ(−CH=CH_2_): 5.08 and 5.22 ppm) show the presence of trace amount of allyl-functionalized polyol chains (structure 7 in Figure 1) in RP5611-10.9, which are formed particularly when the amount of degradation reagent is too low to prevent irreversible thermal degradation of the urethane group to primary amine, olefin, and carbon dioxide;^38^ however, the allyl group has an absorption maximum at a shorter wavelength of light than we used for detection. Therefore, the exact origin of spot 7 remains unknown. Similar to VP5611, the 2D-LC (UV) plot of RP5611-10.9 shows the presence of antioxidant additives at an elution volume of 16.6 mL in SEC dimension, which is outside the *x*-axis region shown in Figure 3.

The LAC × SEC 2D-LC (ELS) plot of RP4811-15.9 shows (Figure 3), similar to RP5611-10.9, high intensity spot 1 corresponding to the fully hydroxyl-functionalized polyol chains. The separation of differently amino-functionalized polyol species and oligomers of RP4811-15.9 in LAC is inferior to that of RP5611-10.9, as shown by 2D-LC, where spots 2 and 3 merge into an asymmetric spot that elutes earlier than spot 1 in the SEC dimension and also show progressively higher molar masses of the later eluting species from the LAC dimension (Figure 3). Nevertheless, the fully hydroxyl-functionalized polyol chains are well separated from all other components of RP4811-15.9, as shown by the 2D-LC recorded by the ELS detector. Therefore, the developed method is still suitable for the determination of the content of fully hydroxyl-functionalized polyol chains in the copolymeric RPs. The presence of oligomers with higher molar mass in RP4811-15.9 compared to those in RP5611-10.9 and their higher content are revealed by SEC/UV-MALS-RI from the lines representing the molar mass as a function of elution volume, UV-signal intensity (Figure S2b), molar mass averages (Table 1), and the intensity of the ^1^H NMR signal (φ) typical for oligomers (Figure S3), all of which is a consequence of the lower degree of degradation of the urethane groups in RP4811-15.9. Similar to RP5611-10.9, the 2D-LC (UV) plot of RP4811-15.9 shows the low-intensity UV-active spots 5 and 7 that co-elute with spot 1. Based on the elution volume of spot 5 in both dimensions, it most likely represents trace amount of fully hydroxyl-functionalized oligomers, while spot 7 is unidentified as in the case of RP5611-10.9.

**Figure 4:**
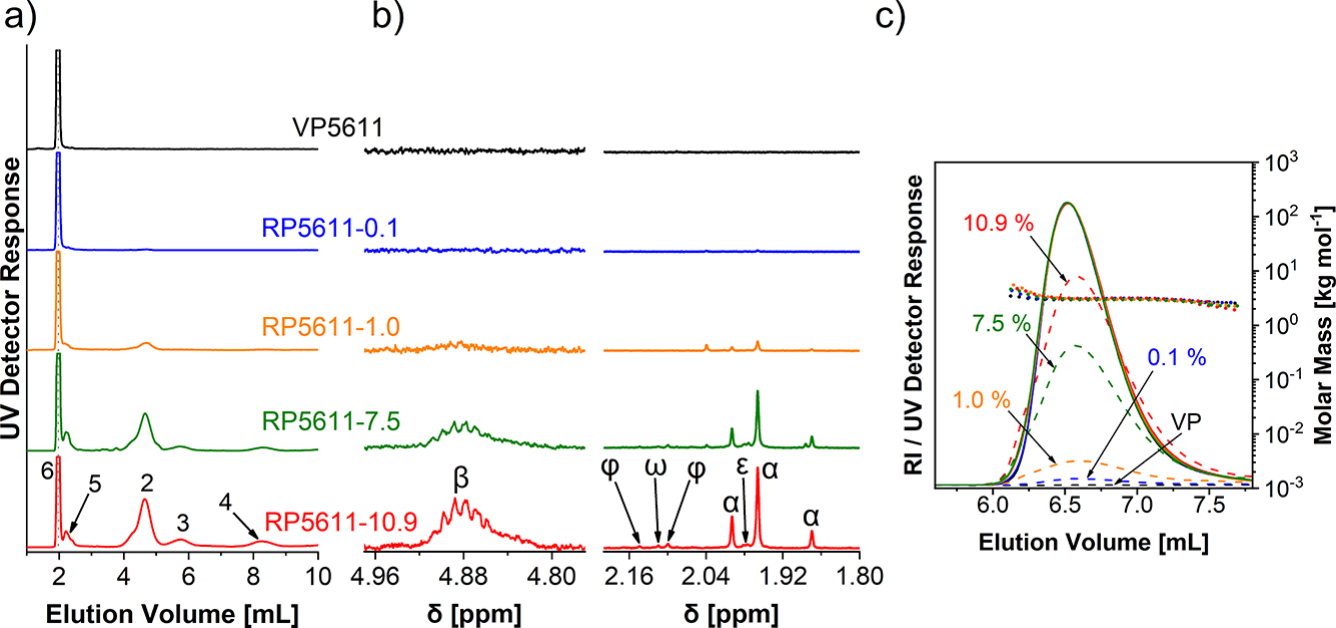
(a) Magnified LAC-UV chromatograms, (b) magnified ^1^H NMR spectra, and (c) SEC/UV-MALS-RI chromatograms of PPO-based VP5611 and corresponding RP5611 samples containing 0.1, 1.0, 7.5, and 10.9 mol % aromatic amino end-groups. (a) Experimental conditions in LAC are the same as described in the caption of Figure 2. (b) ^1^H NMR spectra are normalized to the proton signal of the polyol methyl group. Magnified ^1^H NMR spectra in the δ-range 4.78–4.98 ppm were recorded in DMSO-*d*_6_ with added TFA to shift the overlapping amine end-groups toward the lower magnetic field, while spectra in the δ-range 1.80–2.21 ppm were recorded in DMSO-*d*_6_. For the assignment of the ^1^H NMR signals, see Figure 1. (c) SEC/UV-MALS-RI chromatograms were recorded in MeOH using a TSKgel Alpha-2500 column. The solid and dashed curves represent the RI and UV detector responses, respectively, while the dotted lines show the molar mass as a function of elution volume.

The content of fully hydroxyl-functionalized polyol chains in RPs was determined from peak 1 in LAC-ELS chromatograms of RPs using a calibration curve representing the area under the VP peak (elution volume range: 2.0–4.1 mL) as a function of VP concentration (Figures 2b and S8).^39^ With increasing extent of urethane group degradation (signal β in Figures 4 and S9), the content of fully hydroxyl-functionalized polyol chains increases (Table 1), while the content of oligomers and amino-functionalized polyol chains decreases, as shown by the decreasing intensity of peaks 2–4 in the LAC-UV chromatograms of RPs as well as the decreasing intensity of the UV signal in the SEC chromatograms of RPs and the decreasing intensity of the ^1^H NMR signals corresponding to the amino-functionalized polyol chains (α, ω, ε) and oligomers (φ) (Figures 4 and S9).

The content of fully hydroxyl-functionalized polyol chains in RPs determined by HPLC at low extent of urethane group degradation is higher than the corresponding values determined by ^1^H NMR according to eq S2 because NMR cannot distinguish between mono- and multiamino-functionalized polyol chains, leading to an overestimated content of amino-functionalized polyol chains. Moreover, the molar mass averages of RPs with low extent of urethane group degradation are higher than those of the corresponding VPs, which is due to the presence of oligomers in RPs (Table 1). For higher extents of urethane group degradation (above about 90%), the content of fully hydroxyl-functionalized polyol chains in RPs determined by HPLC agrees well with the content determined by ^1^H NMR, which assumes that RP mixtures consist only of monoamino-functionalized polyol chains and no oligomers in addition to the fully hydroxyl-functionalized polyol chains, and this is true only for the nearly complete degradation of urethane groups (Table 1). In this case, the amount of oligomeric species in the RPs is negligible and the RPs differ from the corresponding VPs only in end-group functionality, which is why the molar mass characteristics of the RPs (weight- and number-average molar masses and dispersity) are comparable to those of the corresponding VPs (Table 1).

## 4 Conclusions

RPs recovered from PUFs by chemical recycling methods after incomplete degradation of the urethane groups in the PUF structure are mixtures of different chain lengths functionalized with terminal amino and hydroxyl groups. The reactivity of amino and hydroxyl groups toward isocyanate groups is particularly different, which significantly affects the relative kinetics of the gelling and foaming processes in the synthesis of PUFs from RPs and ultimately the quality of the PUFs. Therefore, information on the end-group functionality of RPs, as obtained by the HPLC method developed in this work, is important, especially if we take into account that the content of monoamino-functionalized polyol chains in RPs is 3-times higher than the content of amino end-groups of RPs, which means that their contribution cannot be neglected even at a high degree of degradation of urethane groups. Therefore, in this work, we present a simple isocratic and robust HPLC method to separate differently functionalized polyol chains in recycled polyols and to quantify fully hydroxyl-functionalized polyol chains. For this purpose, we use a novel mixed-mode column in combination with organic solvents and ELS and UV detectors. The determination of end-group functionality by HPLC is preferable in view of the availability of HPLC instruments in the industry compared to NMR instruments and the fact that this information cannot be assessed from the hydroxyl number as a conventional parameter for quantification of polyol end-groups.

## Supporting Information Available

Experimental conditions for chemical degradation of PUFs and results of characterization of polyols by ^1^H NMR, HPLC, SEC/UV-MALS-RI, and 2D-LC (PDF)

## References

[ref1] https://www.grandviewresearch.com/press-release/global-polyurethane-foam-market (accessed March 11, 2022).

[ref2] Gama N.; Ferreira A.; Barros-Timmons A. Polyurethane Foams: Past, Present and Future. Materials 2018, 11, 1841–1876. 10.3390/ma11101841. 30262722 PMC6213201

[ref3] Szycher M. Szycher’s handbook of polyurethanes. 2nd ed.; CRC Press Boca Raton: Florida, 2012.

[ref4] Datta J.; Kopczyńska P. From polymer waste to potential main industrial products: Actual state of recycling and recovering. Crit. Rev. Environ. Sci. Technol. 2016, 46, 905–946. 10.1080/10643389.2016.1180227.

[ref5] Datta J.; Włoch M. Recycling of Polyurethanes. In Polyurethane Polymers: Blends and Interpenetrating Polymer Networks, 1st ed.; Thomas S., Datta J., Haponiuk J. T., Reghunadhan A., Eds.; Elsevier: Amsterdam, 2017.

[ref6] Simón D.; Borreguero A. M.; de Lucas A.; Rodríguez J. F. Recycling of polyurethanes from laboratory to industry, a journey towards the sustainability. Waste Manage. 2018, 76, 147–171. 10.1016/j.wasman.2018.03.041. 29625876

[ref7] https://www.marketsandmarkets.com/Market-Reports/polyurethane-foams-market-1251.html?gclid=CjwKCAiAg6yRBhBNEiwAeVyL0Ettn-nusMYl2CSF3AmdJc3l40efb2PHnS8cMcwCLPXIX3t3yBt48xoC0YsQAvD_BwE (accessed 11 March, 2022).

[ref8] Kiss G.; Rusu G.; Bandur G.; Hulka I.; Romecki D.; Péter F. Advances in Low-Density Flexible Polyurethane Foams by Optimized Incorporation of High Amount of Recycled Polyol. Polymer 2021, 13, 1736 10.3390/polym13111736. PMC8198888 34073296

[ref9] Wal H. R. V. New Chemical Recycling Process for Polyurethanes. J. Reinf. Plast. Compos. 1994, 13, 87–96. 10.1177/073168449401300106.

[ref10] Vanbergen T.; Verlent I.; Geeter J. D.; Haelterman B.; Claes L.; Vos D. D. Recycling of Flexible Polyurethane Foam by Split-Phase Alcoholysis: Identification of Additives and Alcholyzing Agents to Reach Higher Efficiencies. ChemSusChem 2020, 13, 3835–3843. 10.1002/cssc.202000949. 32469159

[ref11] Campbell G. A.; Meluch W. C. Polyurethane Foam Recycling Superheated Steam Hydrolysis. Environ. Sci. Technol. 1976, 10, 182–185. 10.1021/es60113a008.

[ref12] Dai Z.; Hatano B.; Kadokawa J.-I.; Tagaya H. Effect of diaminotoluene on the decomposition of polyurethane foam waste in superheated water. Polym. Degrad. Stab. 2002, 76, 179–184. 10.1016/S0141-3910(02)00010-1.

[ref13] Gerlock J.; Braslaw J.; Zinbo M. Polyurethane Waste Recycling. 1. Glycolysis and Hydroglycolysis of Water-Blown Foams. Ind. Eng. Chem. Process Des. Dev. 1984, 23, 545–552. 10.1021/i200026a023.

[ref14] Borda J.; Pásztor G.; Zsuga M. N. Glycolysis of polyurethane foams and elastomers. Polym. Degrad. Stab. 2000, 68, 419–422. 10.1016/S0141-3910(00)00030-6.

[ref15] Wu C.-H.; Chang C.-Y.; Cheng C.-M.; Huang H.-C. Glycolysis of waste flexible polyurethane foam. Polym. Degrad. Stab. 2003, 80, 103–111. 10.1016/S0141-3910(02)00390-7.

[ref16] Molero C.; Lucas A. D.; Rodríguez J. F. Recovery of polyols from flexible polyurethane “split-phase” glycolysis with new catalysts. Polym. Degrad. Stab. 2006, 91, 894–901. 10.1016/j.polymdegradstab.2005.06.023.

[ref17] Nikje M. M. A.; Nikrah M.; Haghshenas M. Microwave Assisted “Split-phase” Glycolysis of Polyurethane Flexible Foam Wastes. Polym. Bull. 2007, 59, 91–104. 10.1007/s00289-007-0753-1.

[ref18] Molero C.; de Lucas A.; Romero F.; Rodríguez J. F. Influence of the Use of Recycled Polyols Obtained by Glycolysis on the Preparation and Physical Properties of Flexible Polyurethane. J. Appl. Polym. Sci. 2008, 109, 617–626. 10.1002/app.28136.

[ref19] Simón D.; García M. T.; Lucas A. D.; Borreguero A. M.; Rodríguez J. F. Glycolysis of flexible polyurethane wastes using stannous octoate as the catalyst: Study on the influence of reaction parameters. Polym. Degrad. Stab. 2013, 98, 144–149. 10.1016/j.polymdegradstab.2012.10.017.

[ref20] Trzebiatowska P. J.; Beneš H.; Datta J. Evaluation of the glycerolysis process and calorisation of recovered polyol in polyurethane synthesis. React. Funct. Polym. 2019, 139, 25–33. 10.1016/j.reactfunctpolym.2019.03.012.

[ref21] Kanaya K.; Takahashi S. Decomposition of Polyurethane Foams by Alkanolamines. J. Appl. Polym. Sci. 1994, 51, 675–682. 10.1002/app.1994.070510412.

[ref22] Chuayjuljit S.; Norakankorn C.; Pimpan V. Chemical Recycling of Rigid Polyurethane Foam Scrap via Base Catalyzed Aminolysis. J. Met. Mater. Miner. 2002, 12, 19–22.

[ref23] Bhuvaneswari G. H. Degradability of Polymers. In Recycling of Polyurethane Foams, 1st ed.; Thomas S., Rane A. V., Kanny K., Abitha V. K., Thomas M. G., Eds.; William Andrew: Norwich, New York, 2018; pp 29–44.

[ref24] Grdadolnik M.; Drinčić A.; Oreški A.; Onder O. C.; Utroša P.; Pahovnik D.; Žagar E. Insight into Chemical Recycling of Flexible Polyurethane Foams by Acidolysis. ACS Sustainable Chem. Eng. 2022, 10, 1323–1332. 10.1021/acssuschemeng.1c07911. 35096493 PMC8790754

[ref25] Molero C.; Ramos M. J.; Lucas A.; Rodriguez J. Chemical recovery of flexible polyurethane foam wastes. WIT Trans. Ecol. Environ. 2010, 140, 69–78. 10.2495/WM100071.

[ref26] Oertel G. Polyurethane Handbook; Hanser Publishers: Munich, Germany, 1985; pp 7–12.

[ref27] Gama N.; Godinho B.; Marques G.; Silva R.; Barros-Timmons A.; Ferreira A. Recycling of polyurethane scraps via acidolysis. Chem. Eng. J. 2020, 395, 125102 10.1016/j.cej.2020.125102.

[ref28] Modesti M.; Simioni F.; Munar R.; Baldoin N. Recycling of flexible polyurethane foams with a low aromatic amine content. React. Funct. Polym. 1995, 26, 157–165. 10.1016/1381-5148(95)00031-A.

[ref29] Li J.; Zhu M. Separation and determination of polyurethane amine catalysts in polyether polyols by using UHPLC-Q-TOF-MS on a reversed-phase/cation-exchange mixed-mode column. J. Sep. Sci. 2018, 41, 831–838. 10.1002/jssc.201700980. 29193805

[ref30] Rissler K.; Künzi H.-P.; Grether H.-J. Chromatographic investigations of oligomeric α,ω-dihydroxy polyethers by reversed-phase high-performance liquid chromatography and evaporative light scattering and UV detection. J. Chromatogr. 1993, 635, 89–101. 10.1016/0021-9673(93)83118-C.

[ref31] Rissler K. High-performance liquid chromatography and detection of polyethers and their mono(carboxy)alkyl and -arylalkyl substituted derivatives. J. Chromatogr. A 1996, 742, 1–54. 10.1016/0021-9673(96)00168-9.

[ref32] Rychłowska J.; Zgoła A.; Grześkowiak T.; Łukaszewski Z. Isolation of poly(propylene glycol)s from water for quantitative analysis by reversed-phase liquid chromatography. J. Chromatogr. A 2003, 1021, 11–17. 10.1016/j.chroma.2003.09.003. 14735971

[ref33] Weidner S. M.; Falkenhagen J.; Maltsev S.; Sauerland V.; Rinkin M. A novel software tool for copolymer characterization by coupling of liquid chromatography with matrix-assisted laser desorption/ionization time-of-flight mass spectrometry. Rapid Commun. Mass Spectrom. 2007, 21, 2750–2758. 10.1002/rcm.3146. 17654465

[ref34] Rissler K. Separation of acetylated polypropylene glycol di- and triamines by gradient reversed-phase high-performance liquid chromatography and evaporative light scattering detection. J. Chromatogr. A 1994, 667, 167–174. 10.1016/0021-9673(94)89064-1.

[ref35] Groeneveld G.; Salome R.; Dunkle M. N.; Bashir M.; Gargano A. F. G.; Pursch M.; Mes E. P. C.; Schoenmakers P. J. Fast determination of functionality-type × molecular weight distribution of propoxylates with varying numbers of hydroxyl end-groups using gradient-normal-phase liquid chromatography × ultra-high pressure size-exclusion chromatography. J. Chromatogr. A 2021, 1659, 462644 10.1016/j.chroma.2021.462644. 34739964

[ref36] https://www.sielc.com/wp-content/uploads/2015/11/SHARC_1.pdf (accessed April 15, 2022).

[ref37] David V.; Grinberg N.; Moldoveanu S. C. Long-Range Molecular Interactions Involved in the Retention Mechanisms of Liquid Chromatography. In Advances in Chromatography, 1st ed.; Grushka E.; Grinberg N., Eds.; CRC Press Taylor & Francis Group: Boca Raton FL, 2017, vol 54; pp 73–110.

[ref38] Ravey M.; Pearce E. M. Flexible Polyurethane Foam. I. Thermal Decomposition of a Polyether-based, Water-blown Commercial Type of Flexible Polyurethane Foam. J. Appl. Polym. Sci. 1997, 63, 47–74. 10.1002/(SICI)1097-4628(19970103)63:1<47::AID-APP7>3.0.CO;2-S.

[ref39] Megoulas N. C.; Koupparis M. Twenty Years of Evaporative Light Scattering Detection. Crit. Rev. Anal. Chem. 2005, 35, 301–316. 10.1080/10408340500431306.

