# Supplementary material for: Determination of End-Group Functionality of Propylene Oxide-Based Polyether Polyols Recovered from Polyurethane Foams by Chemical Recycling
Source: Macromolecules. 2023 Apr 20;56(9):3374–82. doi: 10.1021/acs.macromol.3c00087 (PMC10173687; doi:10.1021/acs.macromol.3c00087)
Supplement: Supplementary file 1 — ma3c00087_si_001.pdf [file ma3c00087_si_001.pdf]

## Supporting Information

# Determination of End-Group Functionality of Propylene Oxide-based Polyether Polyols Recovered from Polyurethane Foams by Chemical Recycling

*Blaž Zdovc,[1] Maja Grdadolnik,[1] David Pahovnik,[1] and Ema Žagar[1,]**

[1]Department of Polymer Chemistry and Technology, National Institute of Chemistry, Hajdrihova 19, Ljubljana SI-1000, Slovenia

*Corresponding author:

Ema Žagar; Email: ema.zagar@ki.si, Phone: +386 1 4760 203

Number of pages: 13

Number of figures: 9

Number of tables: 1

**Table of Contents**

List of Figures ........................................ S2
List of Tables ........................................ S3
Procedure for Chemical Degradation of Flexible Polyurethane Foams (PUFs) ........................................ S4
Determination of the Content of Amino (–$NH_2$) End-Groups in RPs ........................................ S6
Determination of the Content of Fully Hydroxyl-Functionalized Polyol Chains in RPs ........................................ S6
Results ........................................ S8

**List of Figures**

**Figure S1**. Schematic illustration of the on-line coupling of LAC and SEC into a 2D-LC system using an eight-port switching valve equipped with two storage loops to transfer the sample from the first (LAC) dimension to the second (SEC) dimension. ........................................ S7

**Figure S2.** (a) SEC/UV-MALS-RI chromatograms of PPO-based VP5611 and corresponding RPs with aromatic amino end-group content of 0.1 and 10.9 mol %. (b) SEC/UV-MALS-RI chromatograms of P(PO-*co*-EO)-based VP4811 and corresponding RPs with aromatic amino end-group content of 0.2 and 15.9 mol %. The SEC/UV-MALS-RI chromatograms were recorded in MeOH using a TSKgel Alpha-2500 column. Solid and dashed curves represent RI and UV detector response, respectively, while dotted lines represent molar mass as a function of elution volume. ........................................ S8

**Figure S3.** Magnified $^1$H NMR spectra of P(PO-co-EO)-based VP4811 and corresponding purified RPs with aromatic amino end-group content of 0.2 and 15.9 mol %. All $^1$H NMR spectra are normalized to the proton signal of the polyol methyl group. Magnified $^1$H NMR spectra in the δ-range 4.78 – 4.98 ppm were recorded in DMSO-$d_6$ with added TFA to shift overlapping amino end-groups towards the lower magnetic field, while spectra in the δ-range 1.80 – 2.21 ppm were recorded in DMSO-$d_6$. The signal marked with * stands for residual ethyl acetate used for RP purification. The numbers next to the structures refer to the designation of the peaks and spots in LAC chromatograms and LAC×SEC 2D-LC contour plots, respectively. ........................................ S9

**Figure S4.** a) LAC-ELS chromatograms with b) enlarged peaks obtained on a SHARC 1 column at 25 °C for the purified PPO-based RP5611-10.9 with aromatic amino end-group content of 10.9 mol %. The mobile phase composition was 75% ACN with 0.048 vol % MQ and 25% MeOH with 0.1 mg mL$^{-1}$ AmFm, while the amount of FA in ACN was increased from 0.1 to 3.0 vol %. The mobile phase composition for the bottom example was 75% ACN with 3.0 vol % FA and 0.048 vol % MQ and 25% of MeOH with 0.5 mg mL$^{-1}$ AmFm. ........................................ S10

**Figure S5.** a) LAC-ELS chromatograms with b) enlarged peaks obtained on a SHARC 1 column at 25 °C for the purified P(PO-*co*-EO)-based RP4811-15.9 with aromatic amino end-group content of 15.9 mol %. The mobile phase composition was 75% ACN with 0.048 vol % MQ and 25% MeOH with 0.1 mg $mL^{-1}$ AmFm, while the amount of FA in ACN was increased from 3.0 to 6.0 vol %. ........ S10

**Figure S6.** (a) LAC-ELS chromatograms with (b) enlarged peaks and (c) LAC-UV chromatograms with (d) enlarged peaks obtained on a SHARC 1 column at 25 °C for the P(PO-*co*-EO)-based VP4811 and corresponding purified RP4811 samples containing 0.2 and 15.9 mol % aromatic amino end-groups. The mobile phase composition was 75% ACN with 6.0 vol % FA and 0.048 vol % MQ and 25% MeOH with 0.1 mg $mL^{-1}$ AmFm. ........ S11

**Figure S7.** a) LAC-UV chromatograms obtained on a SHARC 1 column at 25 °C for the PPO-based VP5611 and both antioxidant additives. b) LAC×SEC 2D-LC contour plot of VP5611 in the region where antioxidant additives elute. The mobile phase composition in LAC×SEC 2D-LC was in both dimensions 75% ACN with 3.0 vol % FA and 0.048 vol % MQ and 25% MeOH with 0.1 mg $mL^{-1}$ AmFm. ........ S12

**Figure S8.** Calibration curves representing area under the polyol peak in chromatograms of (a) PPO-based VP5611 and (b) P(PO-*co*-EO)-based VP4811 recorded by the ELS detector as a function of VP concentration. The points represent the average peak area of three sequentially injections of the VP solution of the same concentration, while the dashed lines represent the fitted calibration curve. The concentrations of the VP solutions were 1.00, 0.92, 0.84, 0.76, 0.68, 0.60, 0.52, and 0.44 mg $mL^{-1}$. ........ S12

**Figure S9.** a) Magnified LAC-UV chromatograms, b) magnified $^1H$ NMR spectra and c) SEC/UV-MALS-RI chromatograms of P(PO-*co*-EO)-based VP4811 and corresponding RP4811 samples containing 0.2, 6.7, and 15.9 mol % aromatic amino end-groups. a) The experimental conditions in LAC are the same as described in the caption of Figure S6. b) The $^1H$ NMR spectra are normalized to the proton signal of the polyol methyl group. Magnified $^1H$ NMR spectra in the δ-range 4.78 – 4.98 ppm were recorded in DMSO-$d_6$ with added TFA to shift the overlapping amino end-groups towards the lower magnetic field, while spectra in the δ-range 1.80 – 2.21 ppm were recorded in DMSO-$d_6$. For the assignment of $^1H$ NMR signals, see Figure S3. c) The SEC/UV-MALS-RI chromatograms were recorded in MeOH using a TSKgel Alpha-2500 column. The solid and dashed curves represent the RI and UV detector responses, respectively, while the dotted lines show the molar mass as a function of elution volume. ........ S13

**List of Tables**

**Table S1.** Reaction conditions for the degradation of PUFs to produce RPs. ........ S5

**Procedure for Chemical Degradation of Flexible Polyurethane Foams (PUFs)**

The PUFs were synthesized from either a homopolyether polyol (VP5611) or copolyether polyol (VP4811), an isomer mixture of 2,4-TDI and 2,6-TDI with an isomer ratio of 80/20 (TDI index of 107), and water to chemically produce $CO_2$ foaming agent. RPs were recovered from flexible PUFs by microwave-assisted aminolysis under different experimental conditions collected in Table S1.

The one-step aminolysis of PUF5611 was performed with i) 7.25 wt % TREN per PUF5611 (amino per urethane group molar ratio of 1.50) at 220 °C for 30 min; ii) with 4.0 wt % TREN per PUF5611 (amino per urethane group molar ratio of 1.25) at 230 °C for 40 min and iii) with 14.2 wt % PEI-600 per PUF5611 (amino per urethane group molar ratio of 3.80) at 220 °C for 30 min. The obtained RPs are designated as RP5611-10.9, RP5611-7.5, and RP5611-1.0, respectively. The one-step aminolysis of PUF4811 was performed at 180 °C for 30 min with 4.3 wt % TREN per PUF4811 (amino per urethane group molar ratio of 1.50) and with 5.58 wt % TREN per PUF4811 (amino per urethane group molar ratio of 2.00). The obtained RPs are designated as RP4811-15.9 and RP4811-6.7, respectively. The obtained reaction mixtures were centrifuged and the upper polyol phase was poured off and further purified.

RPs with high degree of degradation of urethane groups were obtained by two-step aminolysis of PUFs with TREN. In the first step of the degradation of PUF5611, the reagent was added in an amount of 7.25 wt % TREN per PUF (molar ratio of amino per urethane group of 2.25) and the degradation was carried out at 220 °C for 30 min. Then, the obtained crude RP was subjected to a second aminolysis cycle in which TREN was added in an amount of 2.33 wt % TREN per RP (molar ratio of amino per remaining urethane group of RP of 6) at 220 °C for 20 min. The RP obtained is designated as RP5611-0.1. In the case of two-step aminolysis of

PUF4811, TREN was added in the first step in an amount of 8.30 wt % per PUF (molar ratio of amino per urethane group of 3.00) and the degradation was carried out at 220 °C for 30 min. Then, the obtained crude RP was subjected to the second aminolysis cycle in which TREN was added in an amount of 1.25 wt % TREN per RP (molar ratio of amino per remaining RP urethane group of 6) at 220 °C for 20 min. The RP obtained is designated as RP4811-0.2.

The crude RPs were purified by liquid-liquid extraction using ethyl acetate (EtOAc; c = 1 g $mL^{-1}$) and 0.1 M HCl in water at a ratio of 1/1 followed by pure water to remove low molar mass, amine-functionalized side products (mainly aromatic diamines). EtOAc and water were removed from the purified RPs by rotary evaporation at 60 °C.

**Table S1.** Reaction conditions for the degradation of PUFs to produce RPs.

| *Sample* | *Amine Reagent* | *Amino / Urethane Group* Eqs. | | *Temperature* °C | *Time* min |
|---|---|---|---|---|---|
| | | I. step | II. step | | |
| RP5611-0.1 | TREN | 2.25 | 6 | 220 | 30+20[a] |
| RP5611-10.9 | TREN | 1.50 | / | 220 | 30 |
| RP5611-1.0 | PEI-600 | 3.80 | / | 220 | 30 |
| RP5611-7.5 | TREN | 1.25 | / | 230 | 40 |
| RP4811-0.2 | TREN | 3.00 | 6 | 220 | 30+20[a] |
| RP4811-15.9 | TREN | 2.00 | / | 180 | 30 |
| RP4811-6.7 | TREN | 1.50 | / | 220 | 30 |

[a] Reaction times in the first step and second step of PUF degradation by aminolysis.

## Determination of the Content of Amino (–$NH_2$) End-Groups in RPs

The content of aromatic amino (–$NH_2$) end groups in the RPs was determined from $^1$H NMR spectra of the purified RPs recorded in DMSO-$d_6$ according to Equation S1 from the signal intensities of the aromatic methyl groups (–C$\underline{H}_3$) of the TDA isomer moieties attached to the polyol via the urethane groups (δ (***α***): 2.00, 1.96, and 1.87 ppm for both isomers and δ (***ε***): 1.98 ppm for the aromatic methyl of urea end-group) and the methyl group of the PO repeating unit of the polyol at δ 1.04 ppm, assuming 50 PO repeat units in the polyol arms and polyol functionality of 3. For copolymeric polyols, the content of EO repeating units was taken into account.

$$-NH_2\ content\ (mol\%) = \left(\frac{I(-CH_3)_{\alpha+\varepsilon}\times 50}{I(-CH_3)_{\mathrm{PO}}\times 3}\right)\times 100 \qquad \text{(S1)}$$

## Determination of the Content of Fully Hydroxyl-Functionalized Polyol Chains in RPs

Assuming that RP contains only a single aromatic amino end-group in the structure, the content of monoamino-functionalized polyol chains in RP is three times higher than the content of RP aromatic amino end-groups, since the polyol functionality is three. The content of fully hydroxyl-functionalized polyol chains in RP can be calculated according to Equation S2.

$$Content\ of-OH\ functionalized\ polyol\ chains\ in\ RP\ (mol\%) = 100 - 3\times(-NH_2\ content) \qquad \text{(S2)}$$

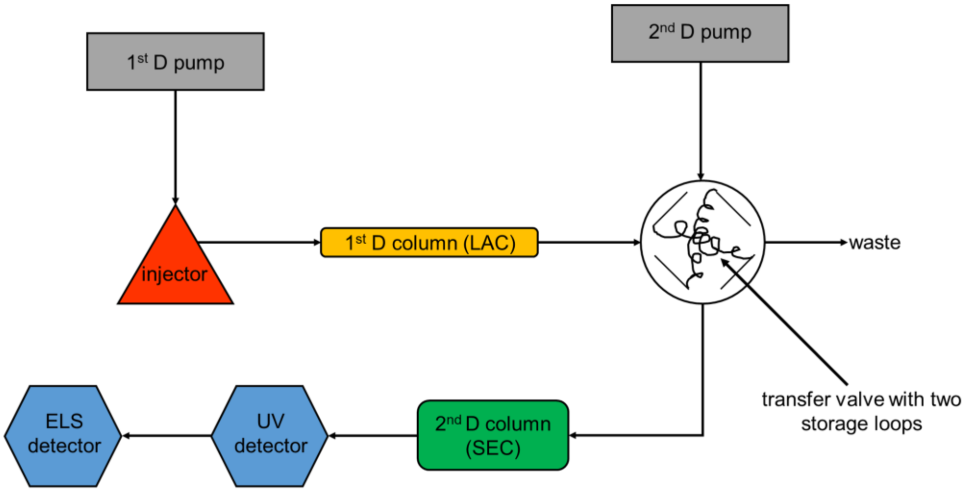


**Figure S1**. Schematic illustration of the on-line coupling of LAC and SEC into a 2D-LC system using an eight-port switching valve equipped with two storage loops to transfer the sample from the first (LAC) dimension to the second (SEC) dimension.

## Results

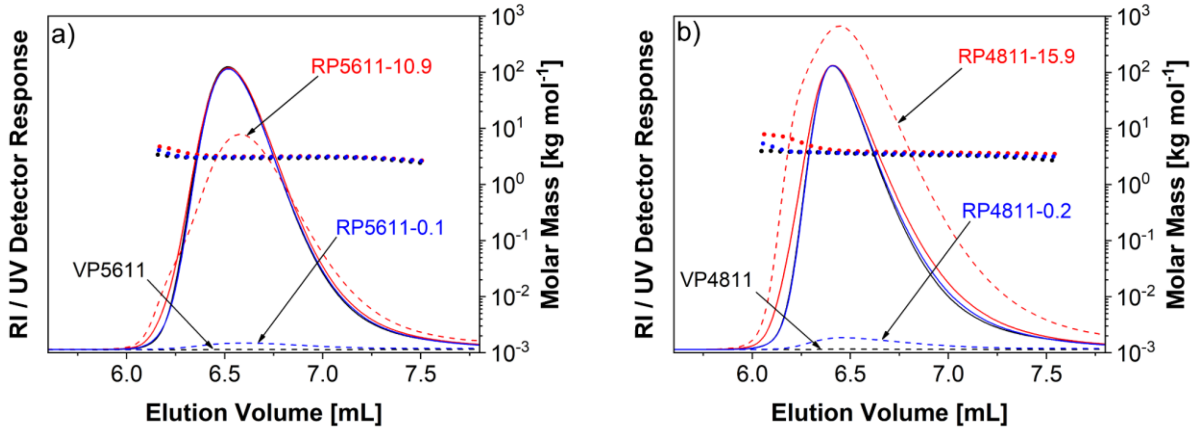


**Figure S2.** (a) SEC/UV-MALS-RI chromatograms of PPO-based VP5611 and corresponding RPs with aromatic amino end-group content of 0.1 and 10.9 mol %. (b) SEC/UV-MALS-RI chromatograms of P(PO-*co*-EO)-based VP4811 and corresponding RPs with aromatic amino end-group content of 0.2 and 15.9 mol %. The SEC/UV-MALS-RI chromatograms were recorded in MeOH using a TSKgel Alpha-2500 column. Solid and dashed curves represent RI and UV detector response, respectively, while dotted lines represent molar mass as a function of elution volume.

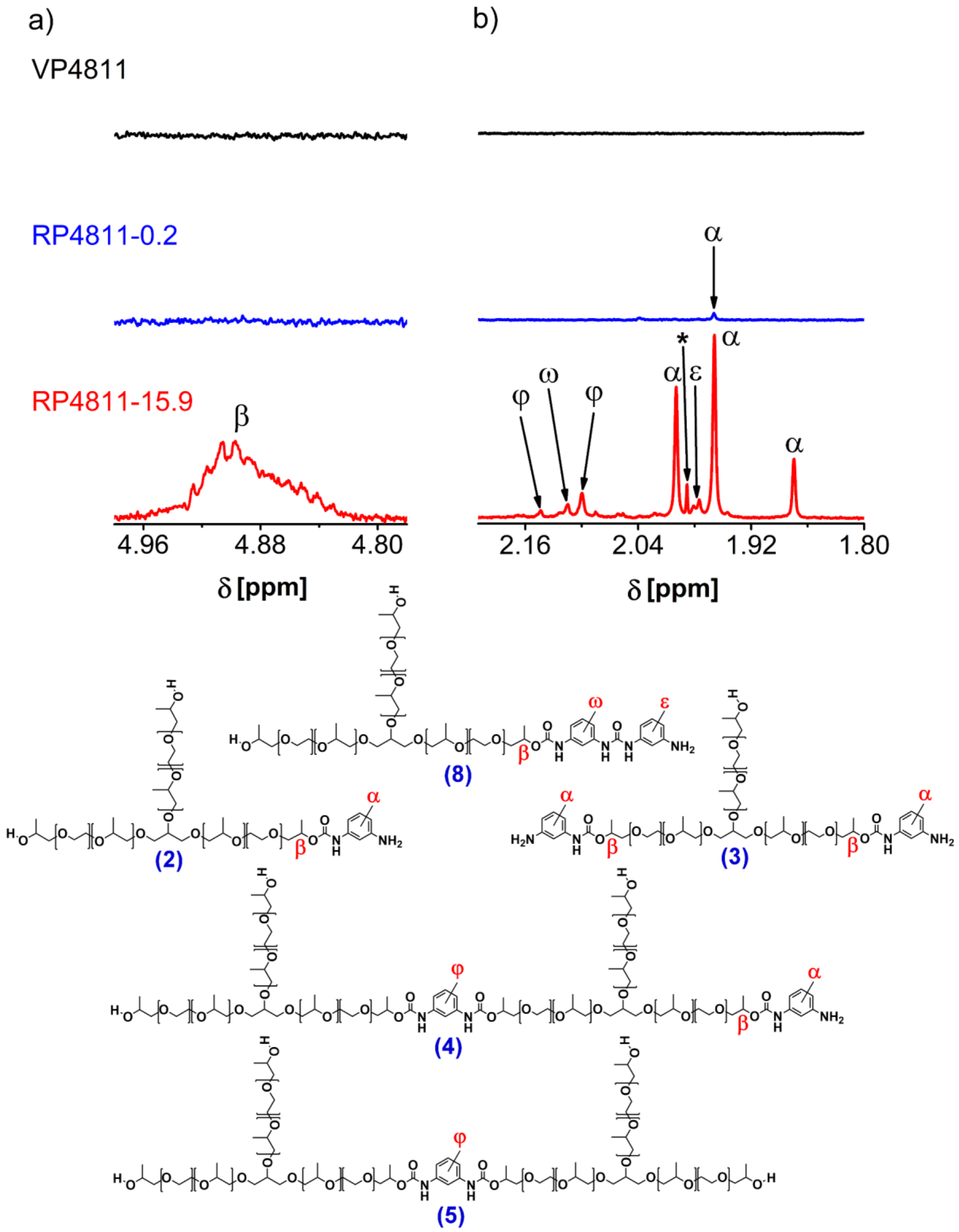


**Figure S3.** Magnified $^{1}$H NMR spectra of P(PO-*co*-EO)-based VP4811 and corresponding purified RPs with aromatic amino end-group content of 0.2 and 15.9 mol %. All $^{1}$H NMR spectra are normalized to the proton signal of the polyol methyl group. Magnified $^{1}$H NMR spectra in the δ-range 4.78 – 4.98 ppm were recorded in DMSO-$d_6$ with added TFA to shift overlapping amino end-groups towards the lower magnetic field, while spectra in the δ-range 1.80 – 2.21 ppm were recorded in DMSO-$d_6$. The signal marked with * stands for residual ethyl acetate used for RP purification. The numbers next to the structures refer to the designation of the peaks and spots in LAC chromatograms and LAC×SEC 2D-LC contour plots, respectively.

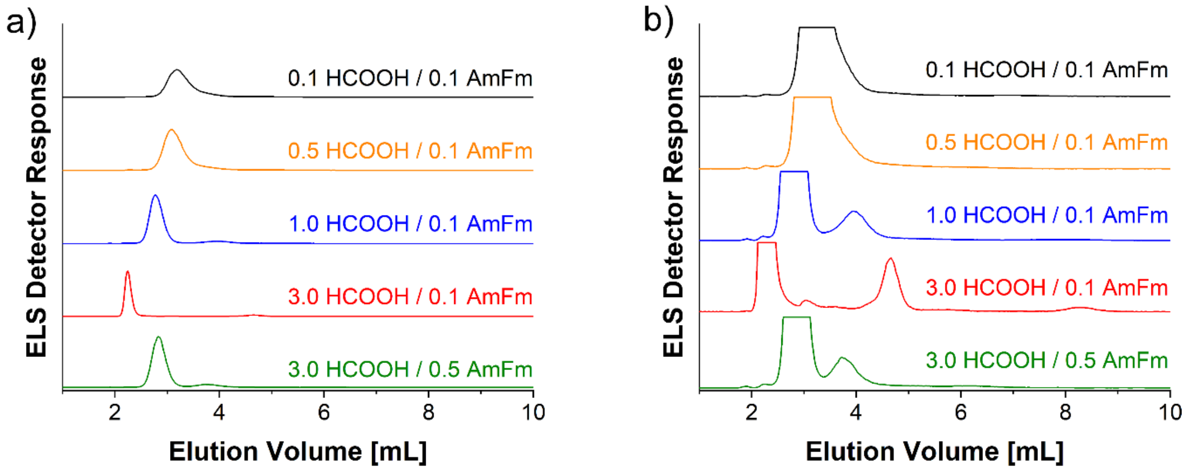


**Figure S4.** a) LAC-ELS chromatograms with b) enlarged peaks obtained on a SHARC 1 column at 25 °C for the purified PPO-based RP5611-10.9 with aromatic amino end-group content of 10.9 mol %. The mobile phase composition was 75% ACN with 0.048 vol % MQ and 25% MeOH with 0.1 mg mL$^{-1}$ AmFm, while the amount of FA in ACN was increased from 0.1 to 3.0 vol %. The mobile phase composition for the bottom example was 75% ACN with 3.0 vol % FA and 0.048 vol % MQ and 25% of MeOH with 0.5 mg mL$^{-1}$ AmFm.

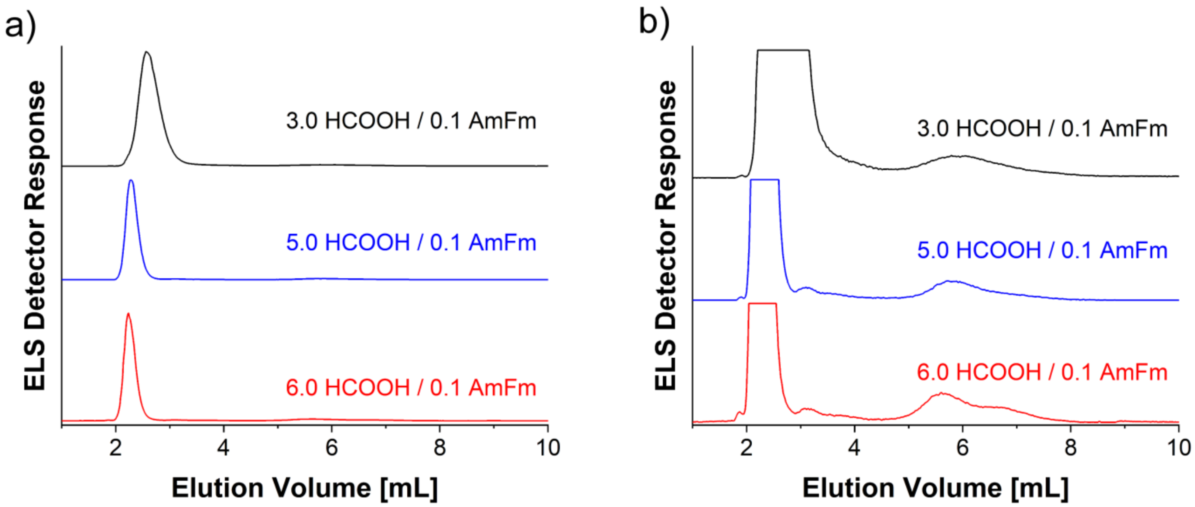


**Figure S5.** a) LAC-ELS chromatograms with b) enlarged peaks obtained on a SHARC 1 column at 25 °C for the purified P(PO-*co*-EO)-based RP4811-15.9 with aromatic amino end-group content of 15.9 mol %. The mobile phase composition was 75% ACN with 0.048 vol % MQ and 25% MeOH with 0.1 mg mL$^{-1}$ AmFm, while the amount of FA in ACN was increased from 3.0 to 6.0 vol %.

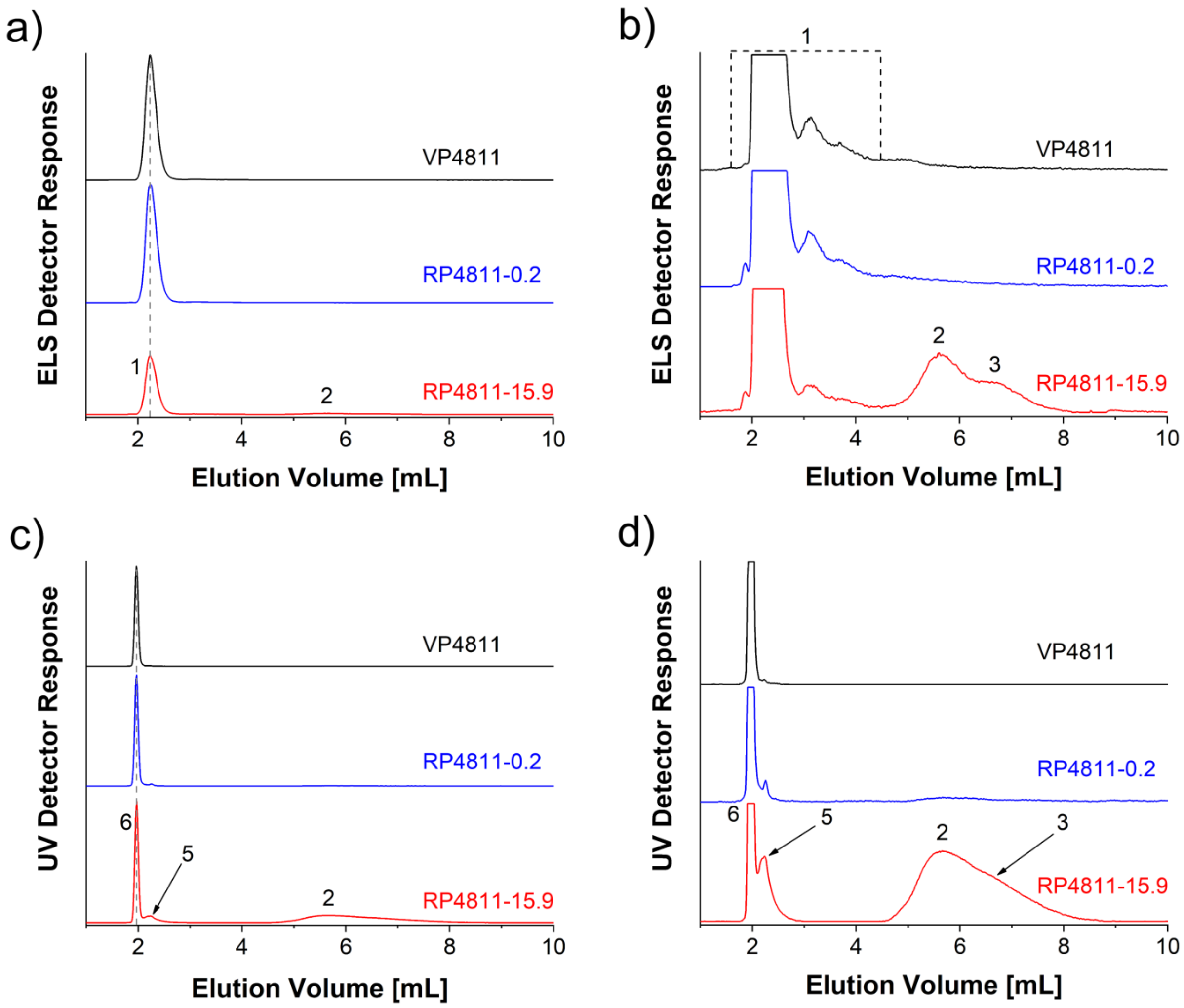


**Figure S6.** (a) LAC-ELS chromatograms with (b) enlarged peaks and (c) LAC-UV chromatograms with (d) enlarged peaks obtained on a SHARC 1 column at 25 °C for the P(PO-*co*-EO)-based VP4811 and corresponding purified RP4811 samples containing 0.2 and 15.9 mol % aromatic amino end-groups. The mobile phase composition was 75% ACN with 6.0 vol % FA and 0.048 vol % MQ and 25% MeOH with 0.1 mg mL$^{-1}$ AmFm.

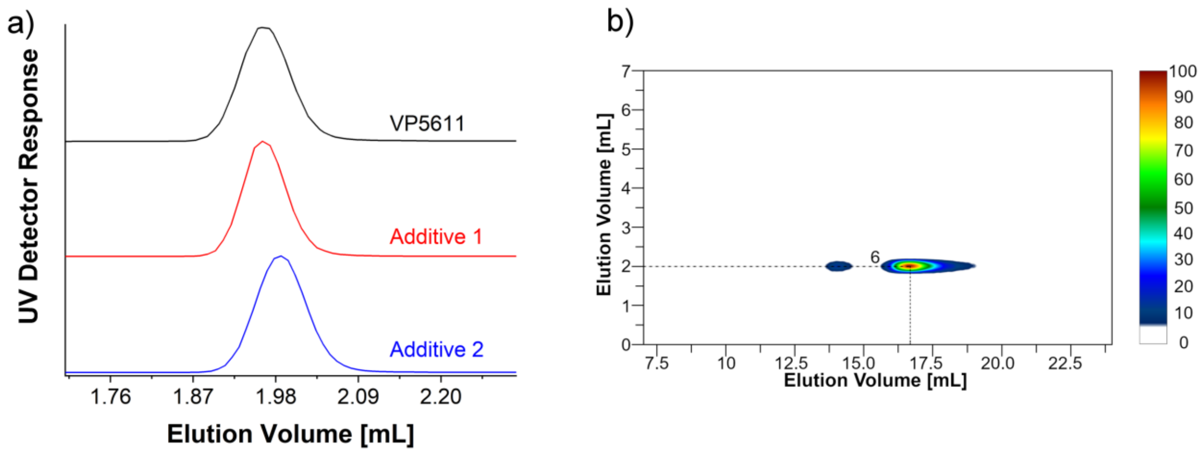


**Figure S7.** a) LAC-UV chromatograms obtained on a SHARC 1 column at 25 °C for the PPO-based VP5611 and both antioxidant additives. b) LAC×SEC 2D-LC contour plot of VP5611 in the region where antioxidant additives elute. The mobile phase composition in LAC×SEC 2D-LC was in both dimensions 75% ACN with 3.0 vol % FA and 0.048 vol % MQ and 25% MeOH with 0.1 mg mL$^{-1}$ AmFm.

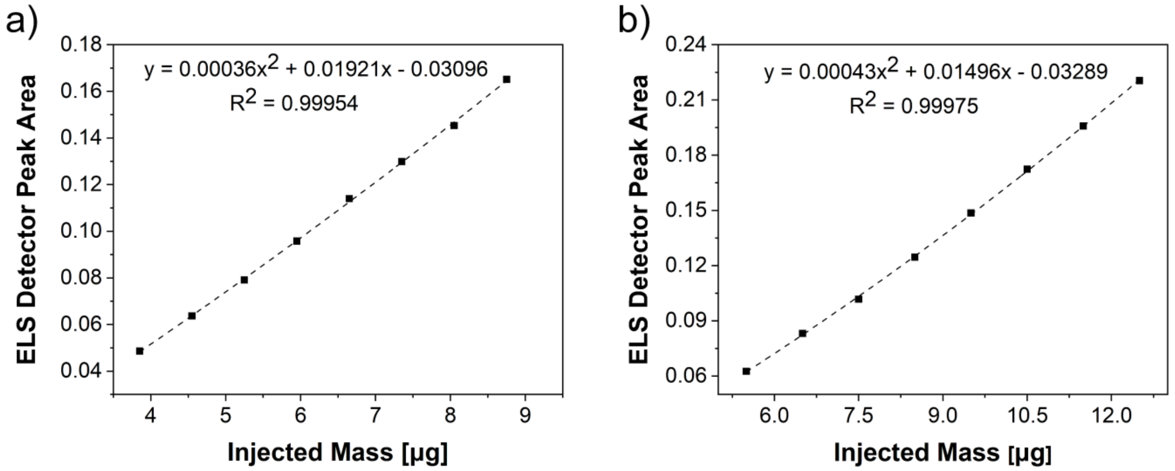


**Figure S8.** Calibration curves representing area under the polyol peak in chromatograms of (a) PPO-based VP5611 and (b) P(PO-*co*-EO)-based VP4811 recorded by the ELS detector as a function of VP concentration. The points represent the average peak area of three sequentially injections of the VP solution of the same concentration, while the dashed lines represent the fitted calibration curve. The concentrations of the VP solutions were 1.00, 0.92, 0.84, 0.76, 0.68, 0.60, 0.52, and 0.44 mg mL$^{-1}$.

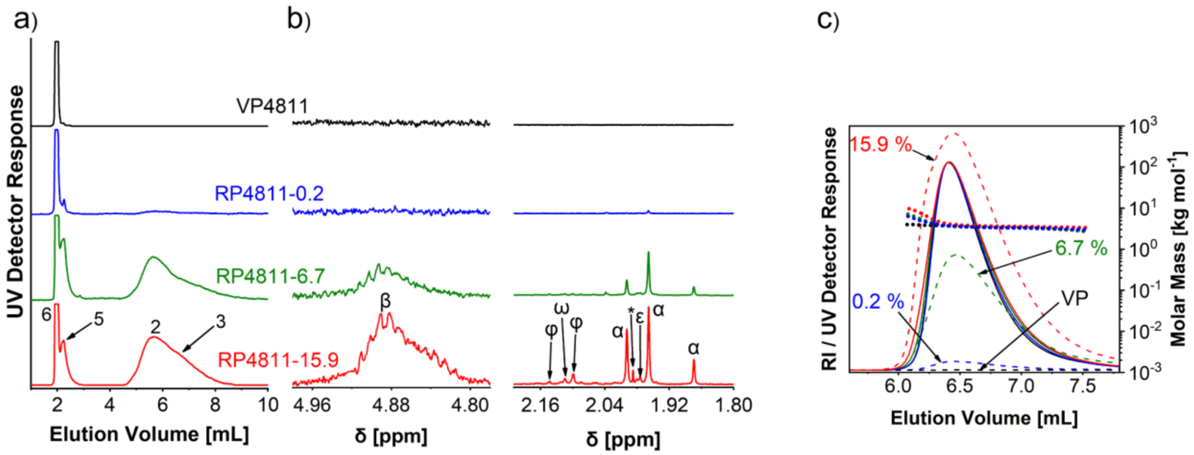


**Figure S9.** a) Magnified LAC-UV chromatograms, b) magnified $^{1}H$ NMR spectra and c) SEC/UV-MALS-RI chromatograms of P(PO-*co*-EO)-based VP4811 and corresponding RP4811 samples containing 0.2, 6.7, and 15.9 mol % aromatic amino end-groups. a) The experimental conditions in LAC are the same as described in the caption of Figure S6. b) The $^{1}H$ NMR spectra are normalized to the proton signal of the polyol methyl group. Magnified $^{1}H$ NMR spectra in the δ-range 4.78 – 4.98 ppm were recorded in DMSO-$d_6$ with added TFA to shift the overlapping amino end-groups towards the lower magnetic field, while spectra in the δ-range 1.80 – 2.21 ppm were recorded in DMSO-$d_6$. For the assignment of $^{1}H$ NMR signals, see Figure S3. c) The SEC/UV-MALS-RI chromatograms were recorded in MeOH using a TSKgel Alpha-2500 column. The solid and dashed curves represent the RI and UV detector responses, respectively, while the dotted lines show the molar mass as a function of elution volume.
